# Association between Maternal Body Composition in Second Trimester and Risk of Fetal Macrosomia: A Population-Based Retrospective Study in China

**DOI:** 10.3390/nu15183879

**Published:** 2023-09-06

**Authors:** Yirong He, Chuanya Huang, Biru Luo, Shujuan Liao

**Affiliations:** 1 Department of Nursing, West China Second University Hospital, Sichuan University, Chengdu 610041, China; he_yirong@126.com (Y.H.); huangchuanya@stu.scu.edu.cn (C.H.); 2 Key Laboratory of Birth Defects and Related Diseases of Women and Children, Sichuan University, Ministry of Education, Chengdu 610041, China

**Keywords:** body composition, bioelectrical impedance analysis, pregnancy, macrosomia

## Abstract

(1) Background: Female body composition undergoes significant changes to support fetal growth and development during pregnancy. This study investigated the association of maternal body composition in the second trimester and macrosomia and explored whether body-composition-related indicators could be used to predict macrosomia. (2) Methods: This study was conducted in China from December 2016 to December 2021. Women with singleton pregnancies, gestational ages between 37 and 42 weeks, and an absence of pregnancy complications were included. In the second trimester, bioelectric impedance analysis (BIA) was used to measure body-composition-related indicators. Logistic regression analysis was performed to explore the risk factors for macrosomia. The predictive performance of maternal body composition and clinical indicators for macrosomia were assessed using the area under the receiver-operating-characteristics curve (AUC). (3) Results: This retrospective study involved 43,020 pregnant women; we collected 2008 cases of macrosomia. Gravidity, gestational age, body mass index (BMI), gestational weight gain (GWG), total body water, fat mass, fat-free mass (FFM), skeletal muscle mass, and visceral fat level were risk factors for macrosomia (*p* < 0.05 for all). In the prediction model, the AUC of FFM for predicting macrosomia was the largest (0.742). (4) Conclusions: Body-composition-related indicators associated with macrosomia and body composition measurements in the second trimester can predict the risk of macrosomia, enabling clinicians to implement interventions earlier to reduce adverse perinatal outcomes.

## 1. Introduction

Fetal macrosomia is a significant problem for neonatal and maternal health, which can lead to a range of serious perinatal complications [1]. The American College of Obstetrics and Gynecology (ACOG) defines fetal macrosomia as an estimated fetal weight or birth weight exceeding 4000 g, regardless of gestational age [2]. Previous studies have shown that the prevalence of macrosomia in developed countries ranges from 5% to 20%, and, in the past few decades, it has increased by 15–25% [3]. Changes in eating patterns and lifestyles have also made macrosomia more prevalent in China as a result of the country’s rapid social and economic development [4]. The prevalence of macrosomia increased from 6.6% in 1996 to 7.0% in 2010 in two hospitals located in the capital city of China, Beijing [5]. According to a retrospective study in 2018, the overall prevalence of macrosomia was 7.2% in China [6]. Macrosomia is linked to a significant risk of both maternal and neonatal complications. Maternal complications related to the delivery of macrosomia include emergency cesarean section, postpartum hemorrhage, anal sphincter injury, while neonatal complications include shoulder dystocia and related problems [7]. Therefore, it is important to explore the risk factors for macrosomia and take corresponding measures to reduce the incidence of macrosomia, which has important scientific and practical implications.

Multiple studies have demonstrated that macrosomia is linked with factors such as genes, environment, race, pre-pregnancy diabetes, delivery history of macrosomia, maternal body mass index (BMI), and parity [8]. Moreover, it is also connected with gestational diabetes mellitus (GDM), gestational weight gain (GWG), fetal sex, gestational age, and other reproductive factors [9]. At present, a large number of studies have focused on the association between maternal BMI, GWG, and delivery with macrosomia. A prospective cohort study in China found that women with overweight or obesity were directly linked to having a higher risk of fetal macrosomia than those with normal pre-pregnancy BMI [10]. According to a large-scale study, GWG beyond the recommendations was related to a higher risk of macrosomia and LGA [11].

In order to prevent adverse pregnancy outcomes, the American Institute of Medicine (IOM) has developed recommended guidelines for weight gain during pregnancy based on women’s pre-pregnancy BMI [12]. BMI is a substitute index to measure the degree of obesity, but the accuracy of BMI as a body-composition marker is controversial. Some researchers have suggested that BMI may be useful to predict body fat indexed to height but not to predict the percentage of body fat [13]. Compared with BMI and other indicators, human body composition can be directly measured from its organizational structure [14]. Human body composition analysis methods include nuclear magnetic resonance imaging technology, dual-energy X-ray detection technology, computerized tomography technology, and bioelectrical impedance analysis [15]. At present, bioelectrical impedance analysis (BIA) is popularly employed in studies on human body composition due to the advantages of its non-invasive, reliable, and fast clinical approach [16]. BIA is used to assess body composition, including fat, protein, total water, and intracellular and extracellular fluid [17].

Female body composition increases dynamically during pregnancy to support fetal growth and development [18]. Changes in body composition are mainly in fat-free body weight and body fat in women in the first and second trimester, to complete most of their body fat reserves [19]. Fat mass is associated with maternal BMI during pregnancy, and increased fat-free mass indicates uterine, placental, and fetal growth. However, current results on the association between maternal body composition and neonatal weight are inconsistent. Therefore, this study aims to explore the body composition of pregnant women in the second trimester through BIA, examine the association between maternal body composition and the risk of macrosomia, and investigate whether body-composition-related indicators could be used to predict macrosomia.

## 2. Materials and Methods

### 2.1. Study Design and Participants

The study was a retrospective study carried out from December 2016 to December 2021 at West China Second University Hospital, Sichuan University. A sample of 43,020 women and their infants who met the following criteria were enrolled in this study: (a) mothers ≥ 18 years old, (b) singleton pregnancy, (c) gestational ages between 37 and 42 weeks, (d) body composition measurements taken in the second trimester, and (e) who had volunteered to participate. Participants were excluded if they (a) had pregnancy complications (i.e., gestational diabetes, gestational hypertension, or intrahepatic cholestasis of pregnancy), (b) fetal growth restriction, (c) intrauterine fetal demise, or (d) fetal chromosomal abnormalities. The study complied with the Declaration of Helsinki and was approved by the Medical Ethics Committee of West China Second University Hospital, Sichuan University.

### 2.2. Measures and Procedure

#### 2.2.1. Body Composition

Body composition measurement was conducted during the second trimester (around 13 weeks gestation) by trained researchers using a professional bioelectrical impedance analysis device (InBody Body Composition Analyzer, BioSpace Co., Ltd., Seoul, Republic of Korea). The InBody is a segmented impedance device that uses eight tactile electrodes to measure voltage drops in the upper and lower body, which uses six frequency bands (1, 5, 50, 250, 500, and 1000 kHz) to measure 30 impedances in five parts of the body [20]. Participants were instructed to wear light clothing and stand barefoot on the metal footplates while holding the hand electrodes. The device measured body impedance, body weight, and multiple indicators related to human body composition. Researchers were uniformly trained before this study started to increase the measurements’ accuracy. All variables were subjected to repeated measurements, and mean values were recorded. In addition, participants were instructed with the following guidelines: no vigorous exercise for 12 h before measurement, no caffeinated foods for 4 h prior to measurement, and consumption of liquids limited to 1% of body weight for 2 h before measurement.

#### 2.2.2. Clinical and Sociodemographic Characteristics

Clinical and sociodemographic data for the pregnant women were retrospectively collected from the hospital’s computerized database, including age, height, reproductive history, past medical history, weight at the time of BIA, gestational age at the time of BIA, and pregnancy complications. Maternal BMI was calculated by dividing self-reported pre-pregnancy weight by the square of the measured height. Gestational weight gain was computed as the disparity between measured weight during BIA examination and self-reported pre-pregnancy weight. Birth weight data were retrieved from hospital perinatal records. Newborns were classified as having macrosomia if their birth weight was greater than 4000 g, regardless of gestational age.

### 2.3. Statistical Analysis

Statistical analysis was conducted by SPSS 26.0 (IBM Corp., Armonk, NY, USA). Descriptive statistics were calculated for clinical and sociodemographic characteristics and body composition measurements. Continuous and categorical variables were presented as mean ± standard deviation (SD) and number (proportion), respectively. The Kolmogorov–Smirnov test (K-S test) was applied to assess the normality of the data, and comparison between (macrosomia vs. non-macrosomia) groups used the two-independent-samples *t*-test, χ^2^ test, or Mann–Whitney U test according to the type and distribution of data. In the univariate analysis of body composition, its relationship with the risk of macrosomia was included in logistic regression analysis. The variance inflation factor (VIF) was used for collinearity diagnosis of the covariates. To evaluate the predictive performance of the risk factors, receiver-operating-characteristics (ROC) curves were generated, and the area under the curve (AUC) was calculated. The optimal cutoff value for each risk factor was determined based on the Youden index, which maximizes the following equation: J = maxc {Se(c) + Sp(c) − 1}, where c represents the cutoff point that yields the highest value for the sum of sensitivity (Se) and specificity (Sp). Following the determination of the cutoff point for each marker, sensitivity and specificity were calculated at the optimal cutoff value. Statistical tests were two-sided, and a *p* value below 0.05 was considered statistically significant.

## 3. Results

**Figure 1:**
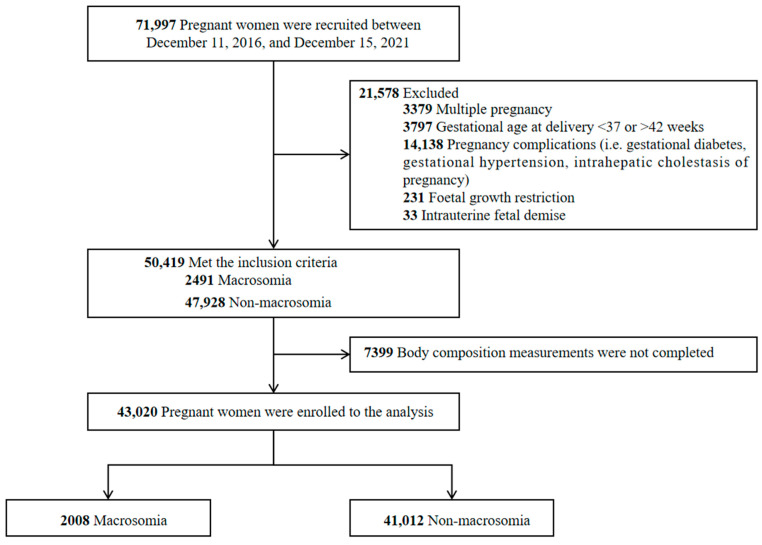
Flowchart of the study participants.

A total of 71,997 participants were included in the study. Excluding the participants who did not meet the inclusion criteria and those who did not complete body composition measurements, 43,020 pregnant women were available for statistical analysis (Figure 1). Table 1 presents the essential characteristics of 43,020 pregnant women, including 2008 who delivered an infant with macrosomia, with a detection rate of 4.67%. The mean age of the participants was 31.27 ± 3.91 years, and the vast majority of participants were Han Chinese (97.94%). Maternal height (*t* = 8.67, *p* < 0.001), weight (*t* = 14.88, *p* < 0.001), BMI (*t* = 12.14, *p* < 0.001), gravidity (*t* = 2.92, *p* = 0.003), gestational age (*t* = 7.61, *p* < 0.001), and GWG (*t* = 16.19, *p* < 0.001) in the macrosomia group were higher than in the non-macrosomia group. Delivery mode (χ^2^ = 249.65, *p* < 0.001) and the incidence of postpartum hemorrhage (χ^2^ = 8.14, *p* = 0.004) were significantly different between the two groups. As for body composition, total body water (*t* = 14.14, *p* < 0.001), protein (*t* = 13.81, *p* < 0.001), minerals (*t* = 13.77, *p* < 0.001), fat mass (*t* = 12.36, *p* < 0.001), soft lean mass (*t* = 14.06, *p* < 0.001), fat-free mass (*t* = 12.36, *p* < 0.001), skeletal muscle mass (*t* = 13.83, *p* < 0.001), bone minerals (*t* = 13.96, *p* < 0.001), percent body fat (*t* = 8.16, *p* < 0.001), waist–hip ratio (*t* = 10.40, *p* < 0.001), visceral fat level (*t* = 10.90, *p* < 0.001), and basal metabolic rate (*t* = 14.11, *p* < 0.001), these exhibited significantly higher levels in the macrosomia group compared with the non-macrosomia group. In contrast, the two groups had no differences in age (*t* = 0.65, *p* = 0.517), ethnicity (χ^2^ = 7.33, *p* = 0.007), parity (*t* = 0.03, *p* = 0.980), or newborn sex (χ^2^ = 0.65, *p* = 0.419).

To investigate the associations between macrosomia and statistically significant factors identified in the univariate analysis, a logistic regression analysis was conducted. Factors exhibiting statistical significance were included as independent variables and macrosomia as a dependent variable (Table 2). In the clinical data, gravidity (OR: 1.096, 95%CI: 1.056–1.138), gestational age (OR: 1.079, 95%CI: 1.067–1.091), BMI (OR: 1.122, 95%CI: 1.074–1.171), and GWG (OR: 1.527, 95%CI: 1.456–1.602) were risk factors for macrosomia. Logistic regression analysis showed that total body water (OR: 2.207, 95%CI: 2.023–2.407), fat mass (OR: 1.023, 95%CI: 1.013–1.033), fat-free mass (OR: 3.068, 95%CI: 2.095–4.493), skeletal muscle mass (OR: 1.062, 95%CI: 1.021–1.105), and visceral fat level (OR: 1.037, 95%CI: 1.022–1.052) increased the risk of macrosomia.

**Figure 2:**
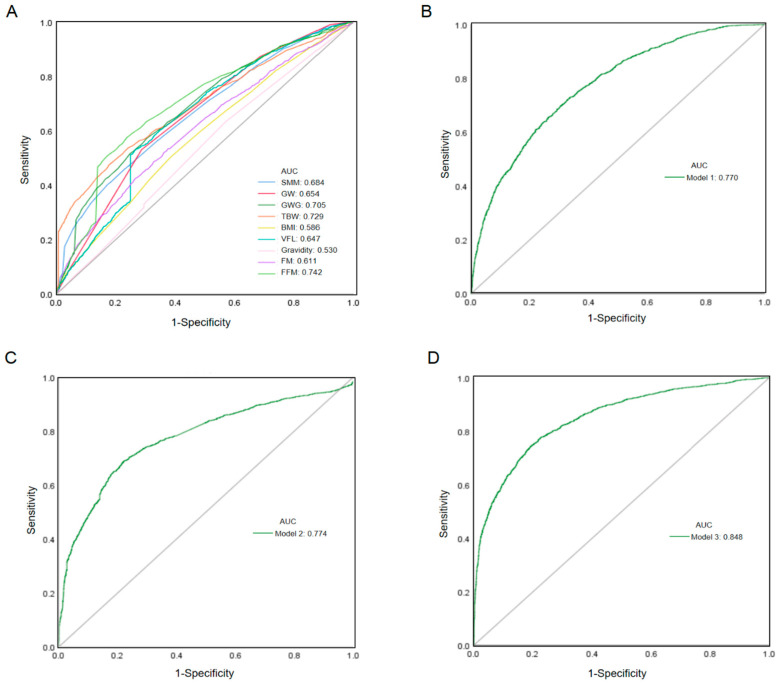
ROC curves to compare the effects of different variables in predicting macrosomia. (**A**) The prediction of macrosomia by individual variables. (**B**) The prediction of macrosomia by model 1. (**C**) The prediction of macrosomia by model 2. (**D**) The prediction of macrosomia by model 3.

The predictive value of macrosomia was analyzed based on pregnant women’s clinical data and body composition measurements. The accuracy of different models and variables for predicting macrosomia were shown in Table 3. Among the clinical factors, the AUC for GWG was 0.705, higher than other risk factors. In the body composition results, the AUC for fat mass in predicting macrosomia was 0.611, the 95%CI was 0.597–0.624, the Youden index was 0.150, and the optimal cutoff value was 19.50; for skeletal muscle mass, the area under the ROC curve was 0.684, the 95%CI was 0.670–0.697, the Youden index was 0.276, and the optimal cutoff value was 23.90. Fat-free mass revealed the highest AUC of 0.742 (95% CI: 0.730–0.755), and its Youden index was 0.336; for total body water and visceral fat level, the areas under the ROC curve were 0.729 and 0.647, respectively. In addition, predictive models for macrosomia based on demographic and clinical data (Model 1), body composition measurement data (Model 2), and the two combined (Model 3) were established. In Model 1, risk factors included the weight of the pregnant women when body composition was measured, gravidity, gestational age, BMI, and GWG. The area under the ROC curve in Model 1 for predicting macrosomia was 0.770 and the 95%CI was 0.758–0.781. Model 2 included measurements of body composition, including total body water, fat mass, fat-free mass, skeletal muscle mass, and visceral fat level. The area under the ROC curve in Model 2 was 0.774 and the 95%CI was 0.761–0.786. Model 3 combined demographic and body composition data, and the AUC was 0.848 and the 95%CI was 0.838–0.858. Receiver-operating-characteristics curves are presented in Figure 2. Collinearity diagnosis is shown in Supplementary Table S1.

## 4. Discussion

This population-based retrospective study investigated the relationship between macrosomia and maternal body composition in the second trimester and established predictive models for fetal macrosomia. This study comprehensively found that weight at the time of body composition measurement, gravidity, gestational age, BMI, and GWG were risk factors for macrosomia. Among the indicators of body composition, total body water, fat mass, fat-free mass, skeletal muscle mass, and visceral fat level were positively correlated with macrosomia. In the predictive model, GWG and fat-free mass had high predictive values for macrosomia. More importantly, the prediction model combining the clinical characteristics and body compositions of pregnant women had the highest predictive performance for macrosomia.

With China’s rapid economic growth over the past four decades, the incidence of macrosomia has also risen. In a study of 84,883 newborns in eastern China, the incidence rates of macrosomia from 1970 to 1979, 1980 to 1989, and 1990 to 1999 were 2.6%, 6.9%, and 13.2%, respectively [21]. A population-based study in Harbin, China, reviewed 13,711 newborns in 16 hospitals between 2001 and 2005, and the prevalence of macrosomia rose from 8.31% to 10.50% [22]. However, since 2010, there has been a decline in the prevalence of macrosomia attributed to alterations in dietary patterns, medical care, and sanitation facilities. A study performed in southern China indicated a reduction in the percentage of macrosomia from 4.0% in 2005 to 2.5% in 2017, representing an annual decline of −4.0% [9]. In rural areas of China’s Henan Province, the incidence rate of macrosomia dropped 31.3% from 8.0% in 2013 to 5.5% in 2017 [23]. In our study, the prevalence of macrosomia was 4.67%. Our study excluded women with gestational diabetes, which is an independent risk factor for macrosomia. Therefore, the incidence of macrosomia in our study was lower than that in other areas in China.

Macrosomia is closely related to genetic factors, the environment, health status, and other maternal factors [24]. Logistic regression results in this study revealed that the risk of macrosomia was significantly and independently associated with gravidity, gestational age, BMI, and GWG. Previous research has demonstrated that pre-pregnancy BMI, gestational excessive weight gain, and existing gestational diabetes/diabetes exhibit metabolic features that are independent risk factors for macrosomia [25,26]. A Japanese study [27] found that maternal age, utilization of in vitro fertilization, labor induction, and gestational age were associated with higher birth weights. Gestational age emerged as the most influential factor impacting birth weight in singleton term infants. In addition, a multicenter cross-sectional study of 101,723 full-term singletons showed a positive correlation between the risk of macrosomia and the mother’s age and gravidity [28].

The composition of the human body includes water, protein, fat, inorganic salts, and other substances, and their relative proportions can offer insights into the body’s nutritional status to a certain extent [29]. Alterations in body composition throughout pregnancy have been linked to adverse outcomes for both the mother and the fetus [18]. This study reported a positive correlation between maternal total body water and macrosomia. Our findings are comparable with prior research in pregnant women that used bioelectric impedance analysis. A longitudinal study conducted in Italy utilized single-frequency tetrapolar bioelectric impedance analysis to assess body composition during pregnancy. Evaluations were initially performed at 15–17 weeks of gestation and subsequently repeated at the second and third trimesters. Body water in the second trimester was predictive of birth weight [30]. This observation can potentially be attributed to the maternal hemodynamic adaptations that occur in the first and second trimesters. It is widely recognized that fetal growth is influenced by the development of low-resistance, high-capacity circulation during these trimesters, which consequently leads to increased total body water and extracellular water [31]. Plasma volume, the major contributor to total body water, has been shown in human and animal studies to correlate with birth weight [32].

Maternal body fat mass, fat-free mass, skeletal muscle mass, and visceral fat level were evaluated, and logistic regression analysis showed that all four factors were risk factors for macrosomia, which was in line with Kent’s study [33]. Newborns’ birth weights were correlated with the maternal content of body fat mass. Body fat mass explained 45% of the variation in birth weight [34]. Maternal fat mass content is likely crucial for the recent increase in birth weight. It is likely that this factor contributes to overall fetal growth rather than specifically stimulating adipose tissue development. Papageorghiou et al. [35] reported that reservation of body fat during pregnancy was mainly concentrated in the first and second trimesters. Reservation of body fat during the first trimester could provide more raw materials to promote fetal growth and development through fat metabolism [36,37]. In addition, Gernand et al. [38] reported that fat-free mass measurements at both 10 and 20 weeks of gestation showed independent associations with birth weight.

Prenatal prediction of macrosomia is essential for clinicians to determine the mode of delivery. Macrosomia was previously predicted by ultrasound and maternal physical examination [39]. Nevertheless, both ultrasonography and clinical measurements have limitations in accurately predicting birth weight [2,40,41]. Hence, it is essential to develop more effective tools to alert healthcare professionals about the potential risks of accelerated fetal growth and macrosomia. As an affordable and practical method, measuring maternal body composition provides a new strategy for predicting fetal macrosomia. In this study, we used BIA to measure the body composition of pregnant women in the second trimester and combine this with clinical data from the pregnant women to predict macrosomia. BIA has been used in various clinical situations and pregnancy conditions, including edema, gestational diabetes mellitus, excessive gestational weight gain, preeclampsia, gestational hypertension, and hyperemesis gravidarum. In a study by Bai et al. [42], excellent test repeatability for BIA throughout pregnancy has been demonstrated. In our study, the predictive model combining body composition with clinical indicators had a high AUC value and greater predictive accuracy for macrosomia compared with standard ultrasound fetal biometry. This predictive model is expected to be used in clinical practice in the future.

The study has several limitations. First, our data did not record information on the history of macrosomia or the dietary intake of the pregnant women during pregnancy, which may increase the risk of macrosomia. If these factors are taken into account, the predictive model may be more effective. Second, due to the impact of ethnic disparities, this study only included the Chinese population, and its findings might not be generalizable to other racial groups. In addition, this study was a retrospective study and only collected body composition data measured in the second trimester, while body composition changed during pregnancy. In the future, prospective longitudinal studies will be conducted to observe the relationship between body composition and birth weight at different points during pregnancy. Finally, there has been a growing body of studies exploring the assessment of fat mass and fat-free mass in pregnant women using bioelectrical impedance analysis. However, bioelectrical impedance analysis may underestimate total body water in the third trimester due to hydration status. Additional validation studies are needed.

## 5. Conclusions

In conclusion, this study comprehensively demonstrated that body-composition-related indicators are associated with macrosomia. Further analysis of the ROC curve revealed that the body composition indicators in pregnant women in the second trimester combined with clinical data had a particular predictive value for macrosomia. In the future, prospective longitudinal studies can be conducted to observe the relationship between body composition and birth weight at various stages of pregnancy so that clinicians can predict the occurrence of macrosomia earlier and implement interventions to reduce adverse perinatal outcomes.

## Figures and Tables

**Table 1:** Participants’ demographic variables, clinical characteristics, and body composition.

Variables	Total (N = 43,020)	Macrosomia Group (N = 2008)	Non-Macrosomia Group (N = 41,012)	t/χ^2^	*p*
Age (years)	31.27 ± 3.91	31.32 ± 3.85	31.26 ± 3.92	0.65	0.517
Nationality				7.33	0.007
Han	42,134 (97.94%)	1974 (98.31%)	40,160 (97.92%)		
Minority	886 (2.06%)	34 (1.69%)	852 (2.08%)		
Height (m)	160.49 ± 4.60	161.41 ± 4.85	160.26 ± 4.51	8.67	<0.001
Weight (kg)	56.83 ± 7.43	59.23 ± 8.32	56.23 ± 7.06	14.88	<0.001
BMI (kg/m^2^)	22.06 ± 2.64	22.80 ± 2.83	21.87 ± 2.56	12.14	<0.001
Gravidity (times)	2.15 ± 1.34	2.20 ± 1.32	2.10 ± 1.33	2.92	0.003
Parity (times)	1.08 ± 0.67	1.08 ± 0.70	1.08 ± 0.66	0.03	0.980
Gestational age	38.87 ± 2.25	39.23 ± 2.28	38.78 ± 2.23	7.61	<0.001
GWG (kg)	14.15 ± 3.69	15.64 ± 3.99	13.77 ± 3.52	16.19	<0.001
Delivery mode				249.65	<0.001
Vaginal delivery	16,872 (39.22%)	450 (22.40%)	16,422 (40.04%)		
Cesarean section	26,148 (60.78%)	1558 (77.60%)	24,590 (59.96%)		
Newborn sex				0.65	0.419
Female	24,392 (56.70%)	1121 (55.83%)	23,271 (56.74%)		
Male	18,628 (43.30%)	887 (44.17%)	17,741 (43.26%)		
Postpartum hemorrhage	1856 (4.31%)	112 (5.58%)	1744 (4.25%)	8.14	0.004
Total body water (kg)	28.36 ± 2.83	29.31 ± 3.13	28.12 ± 2.70	14.14	<0.001
Protein (kg)	7.52 ± 0.76	7.77 ± 0.84	7.46 ± 0.73	13.81	<0.001
Minerals (kg)	2.84 ± 0.28	2.93 ± 0.31	2.82 ± 0.27	13.77	<0.001
Fat mass (kg)	18.12 ± 4.65	19.46 ± 4.99	17.79 ± 4.51	12.36	<0.001
Soft lean mass (kg)	36.34 ± 3.64	37.56 ± 4.02	36.04 ± 3.47	14.06	<0.001
Fat-free mass (kg)	38.72 ± 3.85	40.02 ± 4.27	38.39 ± 3.68	14.11	<0.001
Skeletal muscle mass (kg)	20.69 ± 2.30	21.45 ± 2.54	20.50 ± 2.20	13.83	<0.001
Bone minerals (kg)	2.38 ± 0.24	2.45 ± 0.26	2.36 ± 0.22	13.96	<0.001
Percent body fat (%)	31.44 ± 4.73	32.26 ± 4.52	31.23 ± 4.76	8.16	<0.001
Waist–hip ratio	0.86 ± 0.04	0.87 ± 0.04	0.86 ± 0.04	10.40	<0.001
Visceral fat level	85.90 ± 27.01	92.67 ± 28.62	84.18 ± 26.35	10.90	<0.001
Basal metabolic rate (kcal/day)	1206.39 ± 83.25	1234.38 ± 92.17	1199.32 ± 79.47	14.11	<0.001

Note: age, BMI, gravidity, parity, gestational age, GWG, and body composition are reported as mean (SD). Nationality is reported as n (%). Abbreviations: BMI, body mass index; GWG, gestational weight gain.

**Table 2:** Risk factors associated with macrosomia.

Variables	*B*-Value	SE	Wald χ^2^	*p*-Value	OR (95%CI)
Gravidity (times)	0.091	0.019	22.919	<0.001	1.096 (1.056, 1.138)
Gestational week	0.076	0.006	179.025	<0.001	1.079 (1.067, 1.091)
BMI (kg/m^2^)	0.115	0.022	27.369	<0.001	1.122 (1.074, 1.171)
GWG (kg)	0.423	0.024	304.403	<0.001	1.527 (1.456, 1.602)
Total body water (kg)	0.792	0.044	318.683	<0.001	2.207 (2.023, 2.407)
Fat mass (kg)	0.022	0.005	20.235	<0.001	1.023 (1.013, 1.033)
Fat-free mass (kg)	1.121	0.195	33.148	<0.001	3.068 (2.095, 4.493)
Skeletal muscle mass (kg)	0.060	0.020	8.828	<0.001	1.062 (1.021, 1.105)
Visceral fat level	0.036	0.007	24.545	<0.001	1.037 (1.022, 1.052)

Abbreviations: BMI, body mass index; GWG, gestational weight gain; SE, standard error; OR, odds ratio; CI, confidence interval.

**Table 3:** Accuracy of different models and variables for predicting macrosomia.

Variables	AUC	95%CI	*p*	Cutoff Points	Sensitivity	Specificity	Youden Index
Gravidity (times)	0.530	0.516–0.544	<0.001	4.00	0.632	0.431	0.063
Gestational week	0.654	0.641–0.667	<0.001	37.00	0.530	0.713	0.243
BMI (kg/m^2^)	0.586	0.571–0.600	<0.001	24.50	0.251	0.856	0.107
GWG (kg)	0.705	0.692–0.718	<0.001	14.00	0.397	0.831	0.228
Total body water (kg)	0.729	0.716–0.742	<0.001	37.20	0.474	0.823	0.297
Fat mass (kg)	0.611	0.597–0.624	<0.001	19.50	0.745	0.405	0.150
Fat-free mass (kg)	0.742	0.730–0.755	<0.001	29.90	0.505	0.831	0.336
Skeletal muscle mass (kg)	0.684	0.670–0.697	<0.001	23.90	0.515	0.752	0.276
Visceral fat level	0.647	0.634–0.660	<0.001	85.40	0.510	0.750	0.260
Model 1	0.770	0.758–0.781	<0.001	0.24	0.608	0.780	0.388
Model 2	0.774	0.761–0.786	<0.001	0.22	0.607	0.781	0.388
Model 3	0.848	0.838–0.858	<0.001	0.22	0.737	0.812	0.549

Abbreviations: AUC, area under the curve; CI, confidence interval; BMI, body mass index; GWG: gestational weight gain.

## Data Availability

Not applicable.

## Supplementary Materials

The following supporting information can be downloaded at: https://www.mdpi.com/article/10.3390/nu15183879/s1, Table S1: Collinearity diagnostic statistics for the factors after logistic regression; Supplementary File S1: STROBE Statement.
